# 

**Published:** 1889-03

**Authors:** 


### BOOK NOTICES.

THE CENTURY MAGAZINE for the current month, is, as usual, exceedingly rich in text and illustrations. Those who subscribe for it are more than repaid, and those who do not, are losing many of the good things of this world.

### NEW PUBLICATIONS OF VALUE.

THE NORTH AMERICAN PRACTITIONER, the journal of the Past-Graduate Medical School, of Chicago. Monthly ; $1 a year. Charles Truax & Co., 75 Wabash avenue.

GREELY.—A journal of Natural Science. Monthly. $1.50 a year. M. Wade, publisher, Dorchester, Mass.

### CORRECTION.

There were several errors in the text of Prof. J. R. Buchanan's contribution in our February issue, entitled "The Progress of Hindooism in the United States," which no reader at all acquainted with the author's acquirements would attribute to him. The worst of these errors was wherein the Professor is made to say that he would prefer to be known as a "*ruler*" of divine wisdom, whereas "*seeker*" is the correct word. Other words and phrazes of too common use to afford a reasonable excuse, were misspelled and bedevilled in a most ridiculous way, which, in the hurry of going to press, were uncorrected. Let us be indulgent to the "*poor printer.*"

### NOTE.

We desire to call attention to the article in our current number by a Washington contributor, entitled "The Human Body as a Machine," to be followed next month by a second article quite original in its suggestions concerning the treatment of consumption.

Mr. Harrison is a vigorous writer and thinker and we feel sure our readers will accord to him a hearty welcome.

